# On the shoulders of giants. The story behind the ‘Pioneers of Nephrology’ project

**DOI:** 10.1186/s12882-018-0863-z

**Published:** 2018-03-12

**Authors:** Giorgina B. Piccoli, Bernard G. Jaar, Hayley Henderson

**Affiliations:** 1 Dipartimento di Scienze Cliniche e Biologiche, Università di Torino, Turin, Italy; 2 Nephrologie, Centre Hospitalier du Mans, Le Mans, France; 3 Department of Medicine, Johns Hopkins School of Medicine, Baltimore, MD USA; 4 Welch Center for Prevention, Epidemiology and Clinical Research, Johns Hopkins University, Baltimore, MD USA; 5 Department of Epidemiology, Johns Hopkins Bloomberg School of Public Health, Baltimore, MD USA; 6 Nephrology Center of Maryland, Baltimore, MD USA; 7 BioMed Central, The Campus, 4 Crinan Street, London, N1 9XW UK

## Abstract

This editorial introduces a series of interviews with the pioneers of Nephrology. It’s a story that speaks by itself, given the thousands of people that are now alive thanks to the remarkable advances in renal replacement therapies such as dialysis, and kidney transplantation but also the many scientific advances in our understanding of the pathophysiology and treatment of kidney diseases worldwide.

The interviews that we have selected for this series are, however, not dealing with their achievements, and their success; they try to pass on to future generations the idea of how they were, why they were passionate, what they loved, and, not last, where they found poetry in our profession.

At a time in which narrative medicine points out the importance of the different life experiences in understanding diseases, we would invite you to discover a narrative portrait of the men and women who made our discipline what it is now.

## Introduction

It is extremely difficult to write something about the extraordinary, rich and multifaceted life of the pioneers of Nephrology. It’s a story that speaks by itself, given the thousands of people that are now alive thanks to the remarkable progress with renal replacement therapies such as dialysis, and kidney transplantation but also the many scientific advances in our understanding of the pathophysiology and treatment of kidney diseases worldwide.

Indeed, have you ever tried to stand and write on the shoulder of giants?

Obviously, this is not an easy task, and there are certainly more comfortable positions to write. Nonetheless, that is what we are attempting to do; we are standing on the shoulders of the giants of nephrology. Why then, engage in such an equilibrist’s exercise?

This important and unique project tries to describe the legacy of these nephrology giants, their personality, their challenges, and their successes. Ultimately, this project will inspire all of us to walk in the footsteps of these giants and to emulate their work for a better tomorrow for patients with kidney disease worldwide.

The whole project started almost 10 years ago, on an autumn evening; well, to tell the truth, the whole story started over 30 years ago, in summer, when the corridors of the Nephrology of the “Ospedale Maggiore San Giovanni Battista e della Città di Torino, sede Molinette” were suddenly filled by the warm light of the end of the afternoon.

Since our first encounter at the hospital, Davide and I became friends. Shortly after he was discharged, he introduced me to his good friend Paolo.

Davide was a sculptor, and Paolo was a versatile, multitask artist. For 30 years we have remained friends and have even worked together, running a small art gallery, making projects and writing books. As a result, the Pioneers’ interviews would not have existed without this incredible friendship.

During one of his art travels with his high school students, Paolo discovered the beauty of making movies. We were all engaged in the first one, on the Shoah. It was a work on memory: what was the heritage of Primo Levi, the great writer, who met generations of students, wrote books, fought against the *delitto d’oblio*, and 1 day jumped from the stairway of his home and left this world like a falling angel.

Paolo was not interested in making another film on the Shoah, or on Primo Levi. He wanted to capture what finally became a part of the daily life, a shared memory for his young students.

This was the spirit of the interviews with the pioneers: capturing the permanence of their legacy, not only from the scientific point of view but also considering the way they approached life, as well as clinical problems.

It was while we were dining together, to celebrate Paolo’s movie, and deciding which new adventure to start, that we began thinking about another kind of memory: a history of saving life, the remarkable history of dialysis.

Davide, at that time, had already over 25 years of dialysis. Our initial idea was to tell a novel, maybe his story, maybe a story of a group of people starting their day in different places, and ending up, at the end of the day, in being physicians, patients, nurses….

So, we began plans to create archives of Italian Nephrology and, due to its close relationship, French Nephrology, as a base for our fiction.

At the time, the 50th anniversary of the French Society of Nephrology was nearing, and we thought that their annual meeting could be an opportunity for recording some interviews. We wrote to Professor Patrice Deteix and Professor Pierre Ronco, asking for their permission to record 4-5 interviews, during the meeting in Toulouse. They answered with a list of 15 masters. We proposed a joint venture: we’ll start; if you like what we do, support us to go on further.

Paolo and Davide passed the testimony to Gilberto (Gil) Richiero: (Paolo could not leave school, and Davide was reluctant to change dialysis Centre). Gil and I, therefore, left in my old yellow car, accompanied by a tall, tattooed 18-year-old Siberian film-student that followed Gil like a silent, imposing shadow. We had two enormous cameras and no storyboard. I thought: if we succeed, it’s destiny.

Just before we left, Davide told us: “take the poetry out of them. It has to deal with poetry, in the end”. It was: poetry and destiny.

We listened, fascinated, to Professor Traegers, talking about holding hands when nothing more is left, we heard the story of the father of Professor Ben Mais, who was in Verdun, came back without a leg, and decided that his son had to study not to fight another war, we listened ….

The evening after the first interview, I was gallantly invited to an official dinner. Yves Pirson, with a semi-inquisitive look, asked why we had no storyboard. I answered “*Sir, you do not have a diagnosis before the anamnesis; come tomorrow and see how we work*” and quickly thought we may have made a new enemy. I couldn’t have been more wrong. He came, he weighted, he observed.

A couple of months later, he sent me a concise e-mail, asking how much time we needed to show our film on the pioneers. None of us had any idea about mounting a film; however, during that week in Toulouse, we simply forgot about writing our story. We just wanted to listen to the pioneers’ stories more and more. We made the movie, and it was first shown in Brussels; Gil was with us, as was Paola and his wife, who had also taken up filming and later joined our project. My son, who was also present, commented after seeing the first mounting “*they all smiled, all of them smiled in the end*”.

Then the story went on, thanks to our self-elected producer, Pierre Ronco, thanks to the passion of the pioneers, and to the generosity of Gil and Paola, to the interest of the French Society, and of the ERA-EDTA, also thanks to a few enlightened sponsors. From France and Italy, we moved to the rest of Europe.

Paolo and Davide remained backstage, no less important, souls of this long story. They fell in love with Professor Richet (we all did), were deeply moved by Man “*we are not there to heal, but to ease suffering*”, laughed at the story of how Kokot succeeded in obtaining the papal audience, we admired the beauty of Anita Aperia, the strength of Natalia Tomilina, and the irony of Bernard Rossier.

We traveled through Europe, we moved to the new world, to French-speaking Canada. Over 30 interviews are now mounted, four movies are available on the internet. This is a work in progress, and more interviews are still to come.

We truly enjoyed mounting the history of the nephrology pioneers; we hope that you’ll enjoy and learn from their experience as we did: the joy of life, the sorrow of death, the beauty of science.

We hope to be able to continue to capture the marvelous history of many other nephrology pioneers, and, as in our initial work, we dedicate this experience to the missing ones: dead or forgotten, humble physicians, as well as luminaries: you showed the way, and we thank you.

**Fig. 1:**
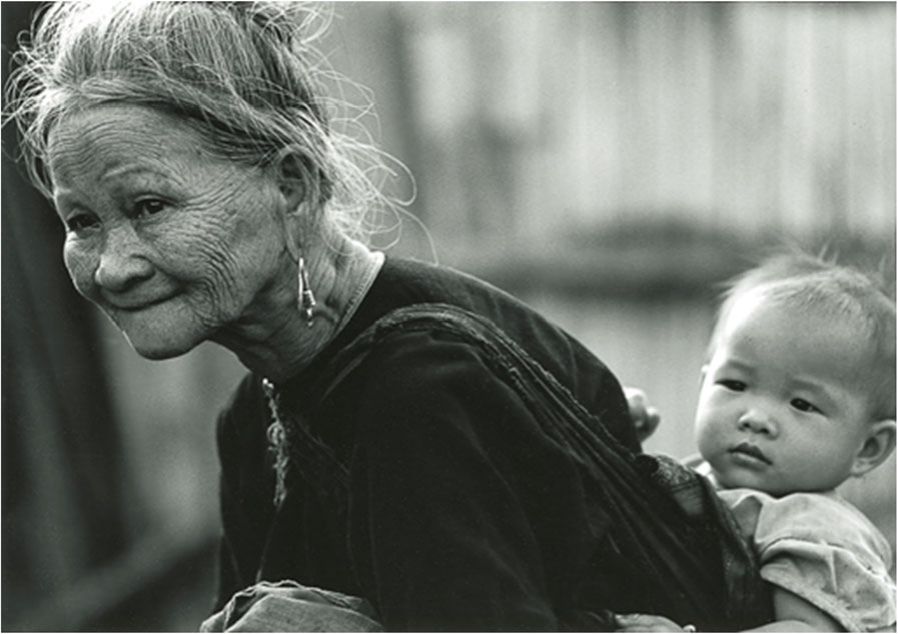
On the shoulders of the giants

Figure 1: Jean Yves Blais is a French voyager and photographer. Curious of human souls, he never depicts people without permission, or without having exchanged with them. Admiration of interior beauty is the driving force of his work; we chose this picture to introduce the project “On the shoulders of the giants”, on the account of his comment on the importance of passing knowledge down through the generations, a concept that could be applied to each art and profession, including medicine.

Permission to use this image has been granted by the photographer, Jean Yves Blais.

